# Ticagrelor-Induced Prolongation of the QTc Interval

**DOI:** 10.1155/2019/5984847

**Published:** 2019-11-07

**Authors:** Avik Ray, Ahmad Najmi, Gaurav Khandelwal, Balakrishnan Sadasivam

**Affiliations:** ^1^Department of Pharmacology, All India Institute of Medical Sciences Bhopal, Saket Nagar, Bhopal 462020, Madhya Pradesh, India; ^2^Department of Cardiology, All India Institute of Medical Sciences Bhopal, Saket Nagar, Bhopal 462020, Madhya Pradesh, India

## Abstract

**Background:**

Ticagrelor has been accepted as a class I antiplatelet agent in patients undergoing percutaneous coronary angioplasty (PTCA). There have been cases reported on ticagrelor being associated with various cardiac conduction defects. But there is no evidence of QTc prolongation associated with the drug as of yet.

**Case Presentation:**

A 64-year-old male who underwent PTCA was given ticagrelor. A baseline electrocardiogram (ECG) showed a QTc of 402 ms. He returned after 1.5 months with complaints of shortness of breath. An ECG revealed a prolonged QTc of 468 ms. Ticagrelor was discontinued in view of ticagrelor-induced dyspnea and the patient was started on clopidogrel. The other medications were kept unchanged. The patient returned after a month without any complaints. A follow-up ECG showed a reduced QTc of 425 ms.

**Conclusion:**

We present a case of ticagrelor-induced QTc prolongation. To our knowledge, this is the first case to be reported on the same. The Naranjo algorithm for causality assessment gave a total score of 6 indicating that the adverse drug reaction falls under the *probable* category.

## 1. Introduction

QTc prolongation is defined as the QTc (QT interval corrected by the RR interval) ≥ 460 ms in men and ≥ 480 ms in women [1], although this definition varies depending on the source. It can lead to the development of torsades de pointes (TdP) which might be associated with episodes of ventricular fibrillation, cardiac arrest and sudden death. The risk of developing TdP increases with QTc intervals being more than 500 ms. Various risk factors for QTc prolongation include bradycardia, structural cardiac defects, females, old age (≥ 65 years), metabolic abnormalities, traumatic brain injury (TBI) and the presence of any QTc prolonging agent [1].

Drug-induced QTc prolongation is the most common reason for the condition [2]. Medicines that increase the interval are proposed to do so by interfering or inhibiting the action of delayed rectifier potassium channels [3]. Several antiarrhythmics, antihistamines, antimicrobials, antidepressants and prokinetic agents are known to cause QTc prolongation [3].

Since the approval of Ticagrelor as an antiplatelet agent by the US Food and Drug Administration (USFDA) on July 20, 2011, there has not been a single recorded case report or evidence of it being associated with QTc prolongation. We hereby report a case of probable ticagrelor-induced QTc prolongation.

## 2. Case Report

A 64-year-old male with a medical history of hypertension, diabetes mellitus and hyperlipidemia and a chronic smoker presented with complaints of chest pain which was sharp, continuous in nature and was aggravated by slight walking or lifting of heavy loads. The patient also had complaints of shortness of breath of Grade 4 according to the Medical Research Council (MRC) Dyspnoea Scale [4]. He had a body mass index (BMI) of 25.8 Kg/m^2^. The baseline HbA1c% value was 10.8 gm%. He underwent coronary angiography at the discretion of the concerned cardiologist. A diagnosis of Acute Coronary Syndrome (ACS) with Non-ST elevation Myocardial Infarction (NSTEMI) with an occlusion of 99% in the left circumflex artery (LCX) was made. Percutaneous Coronary Angioplasty (PTCA) was performed on the same day with the placement of one everolimus eluting coronary stent in the LCX.

**Figure 1:**
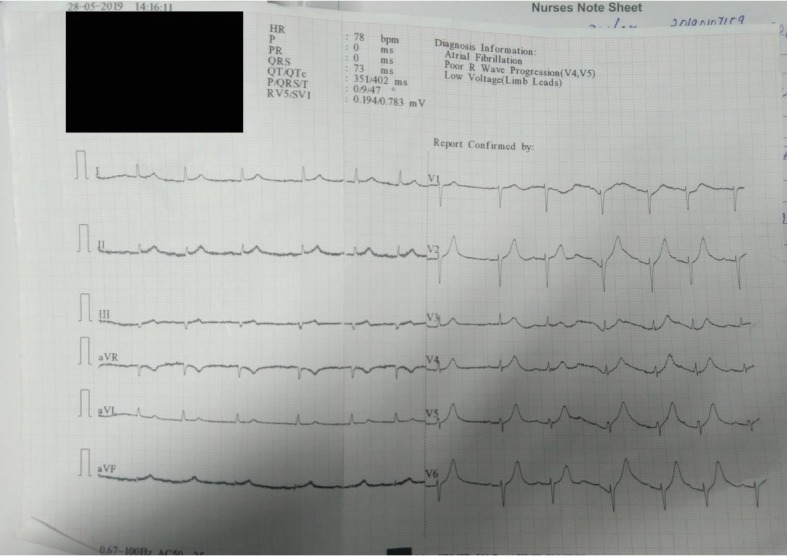
Electrocardiogram showed atrial fibrillation with poor progression of the R wave in leads V4 and V5 along with a QTc of 402 ms.

A loading dose of 180 mg of ticagrelor was given to the patient just before the PTCA was performed. A thrombolysis in myocardial infarction (TIMI) 3 flow was achieved post the procedure. Blood flow distal to the stent was achieved and the procedure was concluded successfully. The post procedural ejection fraction (EF) was 45%. Post procedural electrocardiogram (ECG) was also performed and it showed a QTc of 402 ms with atrial fibrillation and poor progression of the R wave in leads V4 and V5 (Figure 1). He got discharged after 2 days with an advice of 180 mg ticagrelor, 75 mg aspirin, 20 mg atorvastatin, 25 mg metoprolol, 25 mg spironolactone, 10 mg furosemide and 1000 mg of metformin daily. He was advised to visit the out-patient department (OPD) of Cardiology after 2 months or the ED in case of any emergency.

**Figure 2:**
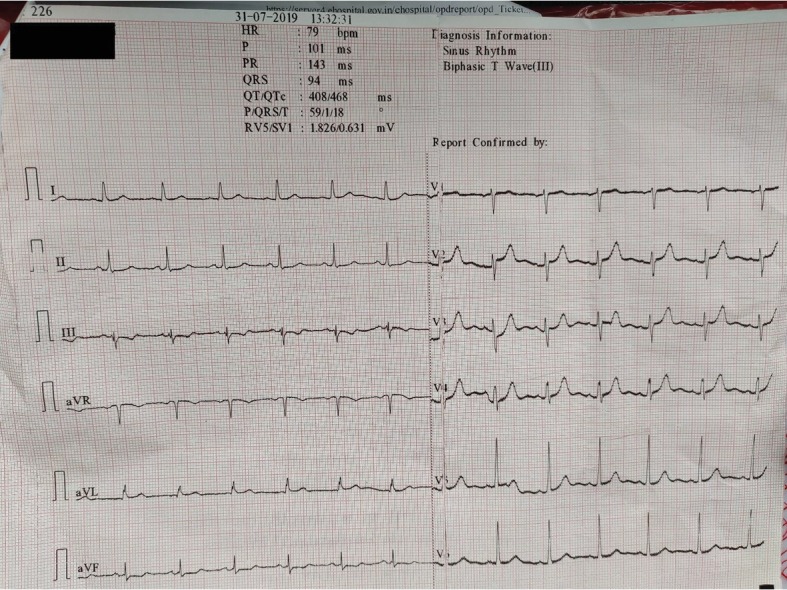
Electrocardiogram revealed a sinus rhythm with biphasic T wave in lead III and a QTc of 468 ms.

The patient came to the OPD after 1.5 months with complaints of severe shortness of breath (MRC Dyspnoea Scale Grade 5) along with infrequent bouts of coughs. Vitals were measured and the blood pressure was 104/60 mm Hg, with the pulse being 68/min. On auscultation, no bronchopulmonary abnormality could be inferred. Bilateral clear breath sounds were obtained. A BMI of 24.6 Kg/m^2^ was recorded. In view of ticagrelor induced dyspnea, ticagrelor was discontinued and 75 mg clopidogrel once daily was started while rest of the medications were kept unchanged. An ECG was obtained and it showed a QTc of 468 ms (16.4% increase) with biphasic T wave in Lead III (Figure 2). He was advised to visit the Cardiology OPD after 1 month or the ED in case of any emergency.

**Figure 3:**
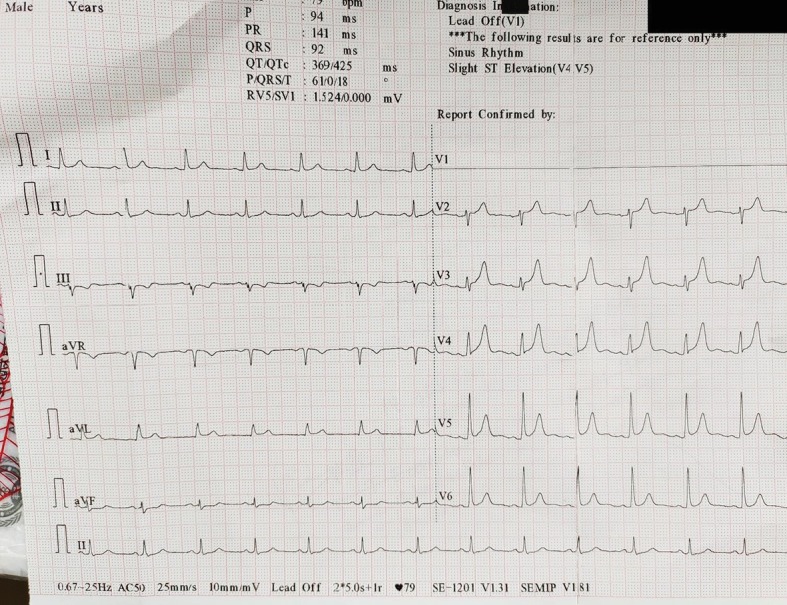
Electrocardiogram revealed a sinus rhythm with slight ST elevation in leads V4 and V5. The QTc was 425 ms, a reduction of 9.2% as compared to the previous reading.

The patient came back after one month for follow-up. He had no complaints of shortness of breath or cough this time. Vitals were measured and blood pressure was 114/78 mm Hg with a pulse rate of 66/min. HbA1c% came out to be 9.6 gm%. An ECG was done and it showed a drastic reduction of the QTc to 425 ms (9.2% reduction) (Figure 3) with slight ST elevation in leads V4 and V5. An echocardiography was also performed and the EF came out to be 50%.

## 3. Discussion

A literature search was conducted to find out any report or evidence of QTc prolongation associated with ticagrelor use. A PubMED search was done using the keywords *ticagrelor*, *QTc prolongation*, *cardiac*, *conduction defects*, *torsades de pointes* and *arrhythmia*.

A single study concluding that a single oral dose of ticagrelor does not prolong the QT interval in health subjects could be found [5]. The association of ticagrelor use with other conduction defects such as atrioventricular block [6, 7] is well known. During a phase IIb dose-ranging safety trial, an unexpected high number of incidences of ventricular pauses was seen in patients receiving ticagrelor [8]. The pivotal trial PLATO monitored for such conduction related adverse events via continuous ECG monitoring on over 3000 patients in the form of a sub-study. Ticagrelor was associated with an increased rate of ventricular pauses ≥3 seconds (5.8% vs. 3.6%, RR: 1.61; 95% CI, 1.14–2.26) [9].

Latest studies have discovered that ticagrelor prevents the uptake of adenosine by the red blood cells in patients of ACS, leading to an increased adenosine plasma concentration (APC), similar to the observation obtained for dipyridamole [10]. Adenosine exerts cardiological effects by acting on the A1 receptors. It has got negative chronotropic effects by suppressing the automaticity of the pacemaker cells and a negative dromotropic effect by inhibiting the atrioventricular bundle [11]. The effect of ticagrelor on the adenosine concentration conveys unique properties to this drug as compared to other P2Y12 antagonists namely clopidogrel, cangrelor and prasugrel.

On performing causality assessment using the Naranjo algorithm [12], a total score of 6 was obtained indicating that the adverse drug reaction falls under the *probable* category (Table 1). The case was reported on the national pharmacovigilance portal.

Other non-pharmacological causes of QTc prolongation such as hypothyroidism [13] and hypocalcemia [14] were ruled out in this patient. As mentioned earlier, the patient was a known case of type 2 diabetes mellitus which has been found to be associated with prolongation of the QTc interval [15]. Since the initial QTc interval was normal and it came back to the normal range after discontinuation of ticagrelor, we can rule out this factor as well. Everolimus has been associated with QTc prolongation in renal transplant patients where it has been used as an immunosuppressant [16]. The drug is usually undetectable in blood 30 days after the implantation of the everolimus-eluting stent. It has been seen that only when a patient has the maximum number of four stents, it is possible to achieve a plasma drug concentration similar to that with oral treatment [17]. This helps us to rule out any possible causality between everolimus and the observed QTc prolongation in this case.

We were hence able to identify a temporal and causal relationship between the initiation and discontinuation of ticagrelor and the development of QTc prolongation. To our knowledge, this is the first case to be reported on the same. The mechanism by which it causes QTc prolongation and whether it differs from the usual mechanism of interference with the action of the delayed rectifier potassium channels and thus extending the duration of phase 3 of myocardial repolarization needs to be investigated [3]. Clinicians should be made aware about this so that cardiac monitoring in the form of an ECG can be considered at the baseline and at the subsequent follow-up visits, especially in patients with multiple risk factors for QTc prolongation.

## Figures and Tables

**Table 1:** The Naranjo algorithm to assess the causality. Total score categories are defined as follows: Adverse Drug Reaction (ADR) is: certain >9; probable 5-8; possible 1-4; unlikely 0.

Question	Yes	No	Do not know	Score
Are there previous conclusive reports on this reaction?	+1	0	0	0
Did the adverse event appear after the suspected drug was administered?	+2	-1	0	+2
Did the adverse reaction improve when the drug was discontinued or a specific antagonist was administered?	+1	0	0	+1
Did the adverse reaction reappear when the drug was re-administered?	+2	-1	0	0
Are there alternative causes (other than the drug) that could solely have caused the reaction?	-1	+2	0	+2
Was the drug detected in the blood (or other fluids) in a concentration known to be toxic?	-1	+1	0	0
Was the reaction more severe when the dose was increased, or less severe when the dose was decreased?	+1	0	0	0
Did the patient have a similar reaction to the same or similar drugs in any previous exposure?	+1	0	0	0
Was the adverse event confirmed by objective evidence?	+1	0	0	+1
Total score				**6**
